# Polyculturalism and Attitudes Toward the Continuing Presence of Former Colonizers in Four Postcolonial Asian Societies

**DOI:** 10.3389/fpsyg.2019.01335

**Published:** 2019-06-07

**Authors:** Allan B. I. Bernardo, Maria Guadalupe C. Salanga, Susana Tjipto, Bonar Hutapea, Aqeel Khan, Susanna S. Yeung

**Affiliations:** ^1^Department of Psychology, University of Macau, Taipa, Macau; ^2^Department of Psychology, De La Salle University, Manila, Philippines; ^3^Faculty of Psychology, Sanata Dharma University, Yogyakarta, Indonesia; ^4^Department of Psychology, Universitas Tarumanagara, Jakarta, Indonesia; ^5^School of Education, Universiti Teknologi Malaysia, Skudai, Malaysia; ^6^Department of Psychology, The Education University of Hong Kong, Tai Po, Hong Kong

**Keywords:** polyculturalism, lay theories of culture, intergroup ideologies, postcolonial socieites, intergroup relations

## Abstract

Polyculturalism is the lay belief that cultures are dynamically interconnected and mutually influencing each other historically and in contemporary times. Belief in polyculturalism is associated with various positive intergroup outcomes in intercultural social contexts, but it has never been studied in relation to intergroup attitudes in postcolonial societies. Two studies with participants from four postcolonial Asian societies (total *N* = 1,126) explore whether polyculturalism will also be associated with positive attitudes toward the continuing presence of former colonizers. The historical colonial experience may be socially represented positively or negatively in different societies, and in this context, the studies inquire into whether current attitudes toward former colonizers are positively associated with the belief in polyculturalism. In two studies (after controlling for belief in multiculturalism, genetic and social constructivist lay theories of race, and national identity) polyculturalism was positively associated with favorable attitudes toward continuing presence of former colonizers in Hong Kong, Macau, and Jakarta, but not in Johor Bahru, Malaysia and Wonosobo, Indonesia. The positive association with polyculturalism was found only in the three societies with a high degree of intercultural contact, where the core beliefs of polyculturalism may be more meaningful. The results are discussed in terms of how intergroup relations between former colonizers and colonized peoples are forms of between-society intercultural contact that are also influenced by intergroup lay theories.

## Introduction

Polyculturalism is the belief that cultural groups are interconnected and mutually influencing each other due to past and present interaction and mutual influences (Rosenthal and Levy, 2010). The belief in polyculturalism can be understood as representing a lay theory of culture (Rosenthal and Levy, 2010, 2012) or an intergroup or diversity ideology (Pedersen et al., 2015) that guides individuals’ cognitive, affective, and behavioral responses in intergroup and intercultural contexts. As a lay theory of culture, polyculturalism assumes that culture is an important part of a person’s social identity, but that culture is not a rigidly bounded construct, and instead, that it is a dynamic concept that changes because of the interconnections and influences among cultures (Morris et al., 2015). Studies have shown how belief in polyculturalism has positive intergroup consequences in both within-society and between-society cross-cultural contact (Rosenthal and Levy, 2013). Historically, there is one form of cross-cultural contact that was pervasive in many parts of the world, and this was in the form of colonization or occupation, whereby European states (and later the United States) established political and economic institutions and structures in countries and territories in Asia, Africa, and South America. Historical cross-cultural contact between the local societies (that comprised different ethnic groups that belong to the geographic region) and the foreign colonizers (who come from mostly European ethnic groups) has not been extensively studied by social psychologists, but postcolonial cross-cultural contact still occurs between many former colonies and former colonizers. As Okazaki et al. (2008) observed, “[t]he colonial past is thus not only a historical legacy but a vivid memory and a lived reality for many contemporary individuals…” (p. 94). In two studies, we explore how polyculturalism relates to attitudes toward the continuing presence of former foreign colonizers in four former colonies in Asia: Hong Kong, Macau, Malaysia (in Study 1), and Indonesia (in Study 2).

### Polculturalism and Intergroup Relations

The concept of polyculturalism can be traced to writing of historians (Kelley, 1999; Prashad, 2001) who have noted how past and contemporary contact and exchanges between cultural groups have shaped the cultural norms and practices of each culture. In psychology, polyculturalism has been studied as a lay theory or belief about how cultures are assumed to be dynamic and mutually influencing each other through historical and contemporary interactions and connections (Rosenthal and Levy, 2010; Morris et al., 2015). Polyculturalism has been differentiated from the lay theory of multiculturalism, which also recognizes the importance of one’s cultural background but emphasizes respecting and honoring cultural differences and the goal of preserving cultural distinctiveness, and appreciating the uniqueness of each culture (Rosenthal and Levy, 2010). This distinction between polyculturalism and multiculturalism has been empirically validated in various studies (Rosenthal and Levy, 2010; Bernardo et al., 2016).

But more importantly, polyculturalism has been shown to predict positive intergroup processes in different contexts involving intercultural contact. Bochner (1982) distinguished between within-society and between-society cross-cultural contact, where the former occurs in culturally heterogeneous societies with many different ethnic groups, and the latter occurs in less intense forms when societies host tourists, overseas students, and contract workers with short-term contracts, but happen in more penetrating ways with immigration. Pertaining to within-society intercultural contact, research on ethnically diverse communities in the United States has shown that belief in polyculturalism is associated with stronger interest in and appreciation of diversity, and with comfort with cultural differences (Rosenthal and Levy, 2010, 2012), with willingness for intergroup contact and friendship intentions with people from different ethnic groups (Rosenthal and Levy, 2016), and positive behavioral intentions and policy attitudes related to Muslim Americans (Rosenthal et al., 2015). Pertaining to between-society intercultural contact, polyculturalism is also associated with more positive attitudes and friendship intentions toward people from foreign countries (Bernardo et al., 2013), toward migrant groups (Rosenthal et al., 2019), less prejudice toward refugees (Healy et al., 2017), and cognitive empathy for actors in intercultural situations (Salanga and Bernardo, 2017).

There is some evidence that endorsement of polyculturalism is associated with positive outcomes in intercultural contexts. For example, among students in an ethnically diverse university, endorsement of polyculturalism was associated with higher academic efficacy, stronger sense of belonging, and less use of unhealthy behaviors to cope with intergroup anxiety (Rosenthal et al., 2016). Endorsement of polyculturalism is also associated with the ability to adapt in culturally diverse environments (Bernardo and Presbitero, 2017) and more favorable views about intercultural expressions of globalization (Bernardo, 2019). There is also experimental evidence that priming the core assumption of polyculturalism results in more positive attitudes toward culture-mixing (Cho et al., 2017) and cultural accommodation (Cho et al., 2018).

Although the empirical work on the intergroup consequences of polyculturalism is still limited, there is clear indication that belief in the connections and mutual influences of cultures relates to more positive intergroup attitudes and relations (Rosenthal and Levy, 2013) and is one of the factors that relates to differences in responses to various forms of cultural contact or mixing (Cheon, 2018). There is a need to continue building on the empirical evidence on intergroup consequences of polyculturalism, particularly in countries outside North America, where the formative empirical work has been established, and also in various types of intergroup relationships.

### Cross-Cultural Contact in Colonial and Postcolonial Societies

The forms of between-society intercultural contact studied in polyculturalism research refer to intergroup processes of people in one culture on the one hand and migrants, refugees, foreign students, and tourists who enter or visit the host culture on the other. The forms of intercultural contact between the colonized and colonizer have similarities with, but also important differences from these forms of between-society contact. One of the similarities is that intercultural contact is between cultural groups with visible distinguishing characteristics such as race, language, and religion. But there are more unique and complex characteristics of contact variables in colonial and postcolonial contexts related to on whose territory the contact occurs and how that relates to relative status/power. In the case of tourists, sojourners, and immigrants, cross-cultural contact occurs in the territory of host society, members of whom typically comprise the numerical majority, and have higher status or power relative to the sojourners and immigrants (but maybe less so in relation to tourists). In colonial societies, cross-cultural contact occurs in the territory of the colonized, who also comprise the numerical majority but have lower status and power because of the political, and economic social arrangements. But in postcolonial societies, status and power relations might vary depending on whether there are still strong vestiges of the former colonial power that persist.

In that regard, postcolonial societies are important contexts within which intergroup perceptions and relations may be studied using more focal social psychological approaches. However, social psychologists have not paid as much attention to these postcolonial societies as such. Okazaki et al. (2008) review of psychology research related to colonialism suggest only two main forms of inquiry: the first relates to the impacts of the colonial experience on individuals (e.g., Varas-Diaz and Serrano-Garcia, 2003; Richards et al., 2005; David and Okazaki, 2006) and on the science and practice of psychology in former colonies (e.g., Enriquez, 1992; Bhatia, 2002; Paranjpe, 2002). In a sense, research on the impacts of colonial experience on individuals may reflect the adverse outcomes of cross-cultural contact between the colonizing and colonized cultures. For example, colonialism in African countries is said to have forced individuals into particular racial/cultural identities and also to separate individuals into race-based group identities (Richards et al., 2005). In some cases, this forced identity resulted in feelings of limited worthiness compared to other groups because of their race, a result that was also observed among Puerto Ricans who question the worth of their group identity as a colonized people (Varas-Diaz and Serrano-Garcia, 2003). However, there is not much theoretical or empirical work in social psychology that directly inquires to intergroup relations and attitudes between citizens of former colonies and their former colonizers.

Many contemporary Asian societies are former colonies and are viable contexts within which attitudes regarding former colonizers could be explored. But any attempt to inquire into intergroup attitudes in postcolonial contexts must consider the vast differences in postcolonial experiences across diverse societies. In some territories like Hong Kong and Macau the colonial governments left less than two decades ago, and there are likely to be stronger, and more visible traces of the colonizers in contemporary society. In the case of Hong Kong, some public opinion suggests that Hongkongers still have very positive attitudes toward their former British colonizers (Trans, 2013; Lhatoo, 2015). But other countries like Indonesia and Malaysia established their independence from the colonial government more than half a century ago, and as such, may have weaker affinities, whether positive or negative, with the former colonizers. Still, some public intellectuals suggest that Malaysians still hold positive attitudes toward their former British colonizers (Musa, 2013) in contrast to the rather negative memories and attitudes that Indonesian people have about their former Dutch colonizers who engaged in mass violence against Indonesians (Bijl, 2012; Luttikhuis and Moses, 2012).

The complex nature of postcolonial relations and attitudes is the subject of social science and cultural studies researchers in these different states and territories. We do not aim to review or summarize this vast body of literature but suggest that social psychologists can also contribute to this area of inquiry. For example, the social representations of history approach (Liu and Hilton, 2005) suggests that people’s collective representations of local and international histories influence local social identities and intergroup processes and relations (Hakim et al., 2015) and collective well-being (Ho et al., 2018). It is very likely that how people in these postcolonial societies represent facets of their colonial history would be associated with various social psychological processes related to their former colonizers (see e.g., Liu et al., 2002).

### The Current Study

In this study we aim to contribute to the empirical evidence on the intergroup consequences of polyculturalism by looking at attitudes toward the continuing presence of former colonizers in postcolonial societies in Asia. We assume that the historical colonial experience could be seen as an exemplar of the connection among cultures, where the local culture and the colonizer’s culture come in contact. However, we wish to clarify at the outset that objective is not to show how the colonial experience influences endorsement of polyculturalism. Our hypothesis is not that the colonial experience makes people in postcolonial societies more likely to endorse polyculturalism. Although that argument (or its opposite) could be made, it is not an argument that we are developing, or that we are testing in the current studies. There are currently no clear theoretical assumptions that propose societal level differences in polyculturalism, or that suggest how societal factors might lead to higher endorsement of polyculturalism in one society compared to another. We are not inquiring into factors that predict individual or societal differences in polyculturalism. Instead, as we explain below, we want to study polyculturalism as a predictor of attitudes related to the former colonizers.

As we assume that the continuing presence of colonizers in postcolonial societies is a specific form of intergroup contact, attitudes toward this continuing presence should be positively associated with belief in polyculturalism. However, unlike other forms of intergroup contact with foreign or other cultures studied in previous research on polyculturalism (e.g., migration, refugees, and international students), historical cultural contact in colonial societies is fraught in specific ways, as the relationship between colonizer and colonized was marked with unequal statuses and subjugation. In postcolonial societies, contemporary representations of the historical contact between colonizer and colonized cultural groups are also likely to be problematic. As such, attitudes toward former colonizers and their continuing presence in postcolonial societies are likely to be negative, yet as we noted earlier (Musa, 2013; Trans, 2013; Lhatoo, 2015), it is possible that these attitudes are positive in some postcolonial societies. Although such attitudes are a specific form of intergroup attitudes toward foreign group, there is some historicity attached to the particular target group of former colonizers. So we extend the general assumption that polyculturalism is positively associated with attitudes toward foreign groups to this specific type of foreign group that has complex historical ties.

For purposes of the current study, we measured attitudes toward exemplars of the continuing presence of the former colonizer and used instructions that framed the questions specifically to indicate that the target group is the former colonizer, and not just any foreign group (see description in section “Materials and Methods”). We propose that the extent to which individuals in the postcolonial societies endorse polyculturalist beliefs should be associated with more favorable attitudes toward the continuing presence of representatives of former colonizers in contemporary society (i.e., businesses, mass media, and migrants). We note that polyculturalism as a lay theory is neutral with respect to the valence of the intercultural connection or influence, thus, it would be interesting to test the hypothesized positive intergroup association with polyculturalism across a number of postcultural societies that may vary in terms of how positive or negative the colonial experience was.

We test this main hypothesis in two studies in four former colonial societies in Asia: Hong Kong, Macau, and Malaysia in Study 1, and Indonesia in Study 2. In testing the hypothesized relationship between belief in polyculturalism, we also tried to statistically control for the possible effects of other relevant beliefs. First, given that polyculturalism and multiculturalism are two lay theories that are known to be related to each other (Rosenthal and Levy, 2010) and that multiculturalism is also associated with attitudes toward different outgroups in intercultural contexts (Tadmor et al., 2012; Yitmen and Verkuyten, 2018), we also measured multiculturalism as a control variable. We also measured national identity or positive thoughts and feelings associated with one’s national group (Crocker and Luhtanen, 1990), although it is not clear how national identity would predict attitudes toward formal colonizers. For some postcolonial societies, the constructed national identity might include positive representations of the colonial past, but for some others, it might include representations of struggles against the colonizers. We also note that there is weak evidence for how national identity relates to attitudes toward outgroups (Leong, 2008; Lyons et al., 2010), and that national identity predicts negative attitudes only under specific conditions (e.g., among groups with high group narcissism, Lyons et al., 2013). Thus, the relationship between national identity and attitudes might vary across different cultural groups in the study, so it was important to measure and control the factor in the analysis. Lay theories of race (Hong et al., 2009) moderate how individuals respond to intercultural situations (No et al., 2008). We distinguish between genetic lay theories of race, which essentializes race and assumes that race is primarily a biological concept, and social constructionist lay theories of race, which assumes that notions of race are derived from historical and social construction processes. Previous research has shown, for example, that individuals who hold more social constructionist beliefs about race are more likely to have trust in intergroup contexts compared to those who hold genetic theories of race (Kung et al., 2018). The two lay theories of race might also relate to attitudes toward the members of the former colonizers as a social group. Finally, we considered other background factors like age and sex, but more importantly those relating the individual’s own intercultural backgrounds. We inquired into whether each individual has family members who are currently traveling or working abroad, and who have already permanently migrated abroad. We also asked how many times they have traveled to other countries. We assume that such factors might influence the individual’s attitudes related to diversity and to other countries and sought to statistically control for these, as well. To summarize, we predict that after controlling for the possible influence of personal intercultural experiences, belief in genetic and social constructivist theories of race, multiculturalism, and national identity, the endorsement of polyculturalism will be positively associated with attitudes toward to the continuing presence of former colonizers.

## Study 1

The participants in Study 1 were from three cities that had been colonial societies up to the 20th century: Macau, Hong Kong, and Johor Bahru. The choice of the three cities was by convenience as researchers from these three cities were part of a cross-cultural research group involved in different investigations. We chose to test the same hypothesis in three postcolonial societies instead of just one to allow for greater generalizability of the findings. We briefly describe the three postcolonial societies in the following paragraphs.

Macau is a Chinese city that was under Portuguese administration since 1557 when the Chinese Ming authorities granted the Portuguese authority to establish a permanent settlement in Macau (Ptak, 1992). Although there have been changes in the official designation of the Portuguese control over Macau, Macau was a Portuguese colony until 1999, when Macau reverted to Chinese governance as a Special Administrative Region of the People’s Republic of China (Hao, 2011). For over four centuries, the Macau people were governed under the Portuguese legal, political, and financial systems. There are visible remnants of that Portuguese colonial period up to this day; Portuguese is still one of the official languages, and Portuguese culture is still evident in the city’s architecture, cuisine, arts, and educational system (Yee, 2001; Hao, 2011).

Hong Kong is another Chinese city that was a European colony since the British occupied Hong Kong island in 1841, and the Xin’an Chinese authorities’ cession of Hong Kong to the United Kingdom under the Treaty of Nanjing in 1842 (Carroll, 2007). Subsequently the terms of British control over Hong Kong were stipulated in various lease agreements, the last of which was a 99-year lease agreement signed in 1898 that included British control over more than 200 small islands surrounding Hong Kong island (referred to as Kowloon and New Territories) (Bard, 2002). At the end of this lease in 1997, Hong Kong reverted to Chinese governance as a Special Administrative Region of the People’s Republic of China. But the British legacy is still quite visible in current Hong Kong society, as the British colonial government established strong legal and financial systems, the civil service, basic, and higher educational systems (Chan, 1997; Lau, 1997).

Malaysia was also a British colony until it declared its independence in 1957, but its colonial past is more complicated because what is now known as Malaysia was actually several sultanates or states that were occupied and/or colonized by either the Portuguese, Dutch, and British at different points from the 16th to 18th centuries in their attempts to establish and control trade in the region (Andaya and Andaya, 2015). In 1826 the British formalized control over most of Malaysia with the establishment of the Straits Settlements; more Malay states were later ceded to British control either as British protectorates or colonies (Andaya and Andaya, 2016). Although, the British interest in the Malay states was initially just economic, similar to Hong Kong, the British colonial government also established economic and legal systems, and other social institutions in Malaysia, but this varied across the different states. Thus, the level of colonialism is higher among states such as Johor, Selangor, and Pahang (even if these states were just protectorates), but less is the states such as Sabah and Sarawak (Lange et al., 2006). For the current study, Malaysian participants were recruited from Johor Bahru, the biggest city in Johor, which experienced a high level of colonialism.

### Materials and Methods

University students from Hong Kong (*N* = 232, *M*_age_ = 20.07, *SD* = 2.28), Macau (*N* = 161, *M*_age_ = 19.21, *SD* = 1.34), and Johor Bahru, Malaysia (*N* = 219, *M*_age_ = 20.51, *SD* = 3.17) participated in the study. The samples were not ethnically diverse; all participants from Hong Kong and Macau were ethnic Chinese, and 91.32% of the Malaysian participants were Malay (the rest were Chinese or mixed). Other demographic details specific to each cultural group are shown at the bottom of Table 1, and the age of the participants indicate that most were born after their cities were no longer under the administration of the foreign colonial powers, with the exception of a few who were infants during the years of the handover of Hong Kong and Macau to China.

**Table 1:** Descriptive statistics for three Asian territories.

	Hong Kong			Macau			Malaysia		
	A	*M*	*SD*	α	*M*	*SD*	A	*M*	*SD*
Attitudes toward colonial presence	0.77	1.71	0.74	0.69	0.89	0.72	0.71	−0.01	1.06
National identity	0.80	4.98	0.87	0.77	4.80	0.80	0.77	5.99	0.90
Genetic lay theory	0.65	3.73	0.82	0.66	3.68	0.78	0.74	4.31	0.75
Social constructivist	0.79	3.64	0.98	0.82	3.54	0.91	0.73	3.54	0.91
Multiculturalism	0.65	4.60	0.74	0.65	4.57	0.61	0.80	4.60	0.74
Polyculturalism	0.81	4.36	0.76	0.72	4.52	0.63	0.79	4.36	0.76
Sample N		232			161			219	
% male		63.40			44.70			16.90	
% with relatives abroad		9.90			86.30			68.50	
% with migrant relatives		31.90			66.50			68.90	
% frequent foreign travel		23.70			68.90			1.80	
Age		20.07	2.28		19.21	1.34		20.51	3.17

Participants from Hong Kong were recruited in two ways; some were recruited through a research participants pool and were given credit toward a course requirement, and others were recruited in different campus and were paid for completing the survey. Participants in Macau were all recruited from a research participants pool and were given credit toward a course requirement, while those from Malaysia were recruited from different classes which gave extra credit for participating in the short survey. All participants were first given general information about the study and the procedures and then asked to indicate whether they were giving their informed consent to participate in the study. Those who gave their written informed consent, completed measures of polyculturalism, lay theories of race, multiculturalism, national identity, and postcolonial attitudes.

The participants in Hong Kong completed the measures in English. For the Macau participants, two bilingual students were involved in the translation, one translated the questionnaires into Chinese written in traditional orthography, and the other student reviewed the translations. Participants from Malaysia completed the measures in Bahasa Malaysia, which was translated from the original scale by a professional translator and a reviewed by a bilingual psychologist. The internal consistency (Cronbach α) of each scale for each of the three samples are reported in Table 1; all α-coefficients were at least 0.65.

*Polyculturalism* (Rosenthal and Levy, 2010) was measured using five items about intercultural exchange and interaction (e.g., “There are many connections between different cultures”). Endorsing polyculturalism goes beyond the recognition of and respect for various racial and ethnic groups as it also indicates awareness that these groups share commonalities, are connected, and exist in a sphere on mutual influence.

*Multiculturalism* (Rosenthal and Levy, 2010) was measured through five items that tap into beliefs about the recognizing and being sensitive to the unique features and differences between cultural groups (“There are boundaries between different cultural groups because of the differences between cultures”).

*The Lay Theory of Race Scale* (No et al., 2008) measured genetic and social constructivist views about race using eight items. Items about genetic beliefs examined participants’ agreement with statements highlight race as a fixed category indicative of psychological qualities (e.g., “A person’s race is something very basic about them and it cannot be changed much”). Items from the social constructivist measure highlighted beliefs about race being arbitrary and are subject to changes as a result of sociopolitical shifts (e.g., “Race does not have an inherent biological basis, and thus can be changed”).

The measures for polyculturalism, lay theories of race, and multiculturalism all use a 6-point scale ranging from 1 (*strongly disagree*) to 6 (*strongly agree*).

*National identity* is measured by adapting the private collective self-esteem and importance to identity subscales of a collective self-esteem scale (Luhtanen and Crocker, 1992). For the current study, the term “social groups” is replaced with “nation” so that the four private-collective-self-esteem items refer to the person’s assessment of how good the nation that one belongs to is (e.g., “In general I am glad to be a member of the nation I belong to”), and the four importance-to-identity items tap into how being a member of a specific nation is an important aspect of one’s self-concept (e.g., “The nation I belong to is important to my sense of what kind of a person I am”). The two adapted subscales combined measure positive evaluation and identification with the national group. Each subscale comprises four items (two reverse scored) rated on a 7-point scale from 1 (*strongly disagree*) to 7 (*strongly agree*).

*The postcolonial attitudes measure* includes 5 items that inquire into participants’ views about how harmful or good they see the influence of the presence of their former colonizers. The items were framed specific to refer to the target group as former colonizers. For example, for the Hong Kong participants, the instructions were

“The British handed over administration of Hong Kong to China in 1997. Since then Hong Kong has been a Special Administrative Region of China. More than 15 years after, there is still a continuing presence of the British in HK. Please think of the specific forms of British presence in Hong Kong indicated in each item. Then please decide whether you think the continuing British presence is harmful, neutral, or good for Hong Kong…”

The items referred to different forms of continuing presence in the followings aspects: use of language, presence of cultural products, businesses, and people, and in the observance of laws and policies. “The continued presence of British people in Hong Kong…” is an example of an item used for the Hong Kong participants. The participants responded using a 7-point scale (−1 = *very harmful*, −3 = *somewhat harmful*, 0 = *neutral*, 1 = *somewhat good*, and 3 = *very good*). Participants were told “We leave it up to you to define harmful or good.”

### Results and Discussion

The descriptive statistics for each sample and for each of the variables measured are summarized in Table 1. In addition, the correlations among the key variables for each sample are summarized in Table 2, which shows similar patterns of correlations between Hong Kong and Macau, but different from Malaysia. Notably, polyculturalism was positively associated with attitudes toward continuing colonial presence in Hong Kong and Macau, but not in Malaysia.

**Table 2:** Correlations among key variables for three Asian samples.

		(2)	(3)	(4)	(5)	(6)
**Hong Kong**						
(1)	Attitudes toward colonial presence	−0.07	−0.01	0.21^∗∗^	0.08	0.24^∗∗^
(2)	Genetic lay theory	–	0.20^∗∗^	0.14^∗^	0.04	0.04
(3)	Social constructivist		–	0.04	0.00	0.07
(4)	Multiculturalism			–	0.25^∗∗^	0.53^∗∗^
(5)	National identity				–	0.29^∗∗^
(6)	Polyculturalism					–
**Macau**						
(1)	Attitudes toward colonial presence	0.06	0.01	0.33^∗∗^	0.22^∗∗^	0.40^∗∗^
(2)	Genetic lay theory	–	−0.08	0.26^∗∗^	0.05	0.13
(3)	Social constructivist		–	0.01	−0.13	−0.09
(4)	Multiculturalism			–	0.12	0.51^∗∗^
(5)	National identity				–	0.16^∗^
(6)	Polyculturalism					–
**Malaysia**						
(1)	Attitudes toward colonial presence	0.01	0.05	0.11	0.05	0.12
(2)	Genetic lay theory	–	0.31^∗∗^	0.34^∗∗^	0.06	0.30^∗∗^
(3)	Social constructivist		–	0.08	−0.08	0.13
(4)	Multiculturalism			–	0.16^∗^	0.69^∗∗^
(5)	National identity				–	0.03
(6)	Polyculturalism					–

*^∗^p < 0.05 and ^∗∗^p < 0.01.*

The main test of the research hypothesis involved hierarchical regression analysis for each territory’s data. In the first step of the regression analysis, continuing colonial presence was regressed to the four control variables (i.e., genetic and social constructivist lay theories of race, multiculturalism, and national identity). Polyculturalism was added to the model in the second step. The results are summarized in Table 3, and these results show that regression model was significant only for Hong Kong and Macau; the regression model did not predict the Malaysian sample’s attitudes toward continuing British presence in Malaysia. We first note that none of the control variables was significantly related to postcolonial attitude, but in Hong Kong and Macau, multiculturalism positively predicted postcolonial attitude. However, this relationship became non-significant when polyculturalism was added to the model, which suggests shared variance between the two intergroup lay theories. More importantly and as hypothesized, polyculturalism positively predicted attitude toward continuing British presence in Hong Kong and continuing Portuguese presence in Macau. In both cases, polyculturalism accounted for a small but statistically significant additional portion of the variance after controlling for the other related variables, but this was not the case in Malaysia.

**Table 3:** Summary of regression analysis predictors of attitudes toward continuing colonial presence in three Asian territories.

	Hong Kong		Macau		Malaysia	
	Model 1	Model 2	Model 1	Model 2	Model 1	Model 2
	β	β	β	β	β	β
Age	−0.02	−0.02	−0.03	−0.06	0.07	0.06
Sex^†^	−0.01	−0.02	0.01	0.03	0.02	0.02
Relatives abroad^††^	−0.03	−0.01	−0.17	−0.17	0.00	−0.01
Migrant relatives^††^	−0.02	−0.01	0.20	0.20	0.06	0.06
Frequent travels^††^	0.06	0.07	−0.13	−0.14	−0.09	−0.10
Genetic lay theory of race	−0.09	−0.08	−0.03	−0.04	−0.06	−0.07
Social constructivist lay theory of race	0.00	−0.01	0.01	0.04	0.08	0.07
Multiculturalism	0.23^∗∗∗^	0.14	0.30^∗∗∗^	0.15	0.12	0.04
National identity	0.02	−0.01	0.15	0.12	0.06	0.08
Polyculturalism		0.18^∗^		0.31^∗∗∗^		0.11
*R*^2^	0.06	0.08	0.21	0.27	0.04	0.05
*F*	1.56	1.94^∗^	4.29^∗∗∗^	5.62^∗∗∗^	0.86	0.89
*df*	9, 219	10, 219	9, 150	10, 149	9, 192	10, 191
Δ*R*^2^		0.02		0.07		0.01
Δ*F*		5.17^∗^		14.14^∗∗∗^		1.23
*df*		1, 218		1, 149		1, 191

*^∗^p < 0.05 and ^∗∗∗^p < 0.001. ^†^Male = 1 and Female = 2. ^††^No = 1 and Yes = 2.*

One possible reason for the different result involving the Malaysian sample might be the fact that Malaysia’s colonial experience ended more than 60 years prior to the date of the current study so that questions referring to the continuing British presence might not be constructed by the respondents in relation to any active representation of the colonial experience. Compare this to Hong Kong and Macau, where public discourses still make references to the British and Portuguese administrative years, which ended about 20 years prior to the date of the study. But even if the presence of British in Malaysia is not seen as a post-colonial phenomenon, one would still expect that polyculturalism would be positively associated with attitudes toward the British, as previous studies show that polyculturalism is positively associated with attitudes toward foreign nationals (Bernardo et al., 2013; Rosenthal et al., 2019).

A second possible reason for the different results with the Malaysian sample relates to whether polyculturalism is a meaningful concept to the Malaysian participants in the study. We note that the Malaysian participants were recruited in Johor Bahru, which is not as multicultural as Hong Kong and Macau. Johor Bahru’s population is ethnically diverse with Malays, Chinese, Indians, and other Malaysian ethnic groups; however, it does not have as high level of cultural diversity as Hong Kong and Macau where there are large portions of the population that are non-local. A study of cultural ideologies in Wonosobo, a culturally homogeneous city in Indonesia (Tjipto and Bernardo, 2019) suggests that the meaning polyculturalism may not be constructed in the same ways in communities that do not have regular direct experiences of intercultural contact; instead notions of multiculturalism and polyculturalism might be abstract notions that are given meaning based on more formal social processes like government policy and school instruction. Although the city of Johor Bahru, Malaysia is not as culturally homogeneous as Wonosobo, Indonesia, it is possible that polyculturalism as an intergroup lay theory is not as meaningful among the participants from the city, which could explain why the Malaysian results diverge from the Hong Kong and Macau results.

## Study 2

The first goal of Study 2 is to provide another test of the hypothesized relationship between polyculturalism and attitudes toward continuing colonial presence in another postcolonial society, hoping to replicate the results in Hong Kong and Macau. The second goal was to test whether the level of intercultural contact in the region explains the different results from Malaysia compared to Hong Kong and Macau. We address these two goals by conducting the same survey in two cities in Indonesia that were also former colonies, but that vary in level of intercultural contact.

Indonesia had a long colonial history under the Dutch (Andaya and Andaya, 2015). The Dutch and other European states had presence in the different areas that are now called Indonesia since the 16th century, but the establishment of the Vereenigde Oost-Indische Compagnie (VOC) in 1800 formalized and accelerated Dutch control over the Indonesian archipelago until the 20th century. Although some parts of Indonesia remained independent (e.g., Aceh, Bali, Borneo, and Lombok), much of Indonesia was under Dutch colonial rule until Indonesia proclaimed independence in 1945 (Ricklefs, 1991). Thus, the participants in Indonesia and Malaysia are more similar to each other in the sense that the colonial experience was a more temporally distant experience for them, compared to the participants in Hong Kong and Macau. But the participants in Study 2 were recruited in two cities that vary in level of intercultural contact: Jakarta and Wonosobo. Jakarta is the capital city which is Indonesia’s most urban city and where there is more intercultural contact among the local residents and foreigners (tourists, foreign workers, etc.). Wonosobo is a rural mostly agricultural city where local residents do not have much actual contact with Indonesians from other states much less intercultural contact with foreigners. We recognize that there are other societal differences between the two cities, but for purposes of the study, the cities were selected for the differences in their level of intercultural experiences.

Intergroup lay theories like polyculturalism are assumed to be chronically accessible to individuals in societies where the core propositions of the lay theory are frequently activated (Hong et al., 2001). In such cases, the intergroup lay theory has structural and functional properties that make it theory-like. Structurally, the lay theories are organized knowledge structures comprising of beliefs; a lay theory is not an isolated belief. Functionally, the organized knowledge structures guide perceptions, understanding, inferences, and other social cognitions. In this regard, we propose that the lay theory of polyculturalism is more meaningful in societies with more intercultural contact, as the core ideas associated with similarities, connections, and mutual influences among cultures are more accessible in public discourses. But in societies will less intercultural contact, polyculturalism might be constructed as an isolated belief or proposition and will not have the theory-like function. This difference in how meaningful or theory-like polyculturalism is across societies may or may not manifest in the degree to which the belief is endorsed in different societies. On the one hand, we could expect that in high intercultural contact societies, polyculturalism would be more highly endorsed than in low intercultural contact societies. But on the other hand, endorsement of polyculturalism in low intercultural contact societies could also be high but reflecting endorsement of the isolated belief and not of the organized knowledge structure; as such, comparing mean endorsement may not reflect the actual differences. We propose that the better indicator of whether polyculturalism is meaningful and theory-like in one society is its functional properties, or its association with the expected intergroup outcomes.

Based on these theoretical assumptions, we hypothesized that polyculturalism will also be positively associated with attitudes toward continuing presence of Dutch people, businesses, organizations, and so on, in Indonesia, but that this positive association would only be observed in participants from Jakarta, where polyculturalism will be a more meaningful construct due to the experience of intercultural contact therein. We also hypothesized that there will be no relationship between polyculturalism and the same attitudes toward the Dutch among the Wonosobo participants, for whom polyculturalism will not be a meaningful construct. We did not hypothesize a difference in endorsement of polyculturalism between the two groups, but we tested for this difference to see how it may inform our broader hypothesis.

### Materials and Methods

Five hundred fourteen Indonesian students from the city of Jakarta (*N* = 244) and the regency of Wonosobo (*N* = 270) participated in the study. The participants were recruited from universities in the two cities who were given extra credit in a psychology or a social science class for completing the survey. As in Study 1, only participants who gave their informed consent were given the study questionnaire. Some demographic details specific to each sample are shown at the bottom of Table 4.

**Table 4:** Descriptive statistics for two Indonesian cities.

	High intercultural contact: Jakarta			Low intercultural contact: Wonosobo		
	α	*M*	*SD*	α	*M*	*SD*
Attitudes toward colonial presence	0.69	0.70	0.91	0.67	−0.03	0.98
National identity	0.85	5.34	0.95	0.85	5.84	0.95
Genetic lay theory	0.66	3.88	0.85	0.65	4.06	0.90
Social constructivist	0.78	3.13	0.98	0.79	3.02	0.98
Multiculturalism	0.59	5.03	0.51	0.52	4.98	0.50
Polyculturalism	0.68	4.74	0.61	0.69	4.57	0.74
Sample N		244			270	
% male		68.40			46.00	
% with relatives abroad		45.50			48.10	
% with migrant relatives		60.20			57.80	
% frequent foreign travel		13.90			0.00	
Age		19.80	1.55		19.05	1.28

Indonesian students completed the same set of measures in Study 1. For the Indonesian participants, the measures were translated from English to Bahasa Indonesia by a professional translator and reviewed by two bilingual psychologists. The internal consistency coefficients of the scales are also reported in Table 4. We note that the multiculturalism scale had inadequate internal consistency in both samples; nevertheless, the multiculturalism scores were still included as control variables in the analysis to be consistent with Study 1.

### Results and Discussion

The mean scores for all the key variables for the two Indonesian samples are summarized in Table 4. Although we did not hypothesize about the group differences in polyculturalism, we nevertheless tested whether there was a difference associated with the different levels of intercultural contact in the two groups. The ANOVA indicated that there was a statistically significant difference, *F*(1,511) = 8.23, *p* = 0.004, partial η^2^ = 0.016; the participants from Jakarta endorsed polyculturalism slightly more than their Wonosobo counterparts. But the more interesting test relates to whether polyculturalism was associated with different intergroup variables in the two groups, and looking at the correlations among the key variables also summarized in Table 5, we see different patterns of relationships between the Jakarta and Wonosobo samples. As expected, polyculturalism was positively related with attitudes toward continuing Dutch presence among the Jakarta participants, but not among the Wonosobo participants.

**Table 5:** Correlations among key variables for two Indonesian samples.

		(2)	(3)	(4)	(5)	(6)
**High intercultural contact: Jakarta**						
(1)	Attitudes toward colonial presence	−0.07	−0.04	0.05	−0.09	0.16^∗^
(2)	Genetic lay theory	–	−0.07	0.13^∗^	−0.01	0.01
(3)	Social constructivist lay theory		–	−0.09	0.04	0.11
(4)	Multiculturalism			–	0.16^∗^	0.19^∗∗^
(5)	National identity				–	0.02
(6)	Polyculturalism					–
**Low intercultural contact: Wonosobo**						
(1)	Attitudes toward colonial presence	−0.07	0.03	−0.02	−0.05	0.09
(2)	Genetic lay theory	–	0.05	0.20^∗∗^	0.08	0.09
(3)	Social constructivist lay theory		–	−0.01	−0.06	0.09
(4)	Multiculturalism			–	0.04	0.26^∗∗^
(5)	National identity				–	0.02
(6)	Polyculturalism					–

*^∗^p < 0.05 and ^∗∗^p < 0.01.*

The main test of the hypotheses involves hierarchical regression analyses similar to Study 1. The results are summarized in Table 6. As hypothesized, polyculturalism positively predicted attitudes toward continuing Dutch presence in the Jakarta sample, where the regression model accounted for a small but significant portion of the total variance in the data. But in the Wonosobo sample, the regression model was not significant, and polyculturalism was not positively associated with attitudes toward continuing Dutch presence. These results provide some evidence for the notion that polyculturalism’s positive intergroup consequences is premised on the activation of the core ideas related to the mutual influence and connections of cultures within social environments where there are sufficient levels of intercultural contact. This point is elaborated in the General Discussion below.

**Table 6:** Summary of regression analysis predictors of attitudes toward continuing Dutch presence in two Indonesian cities.

	High intercultural contact: Jakarta		Low intercultural contact: Wonosobo	
	Model 1	Model 2	Model 1	Model 2
	β	β	β	β
Age	0.20^∗∗^	−0.21^∗∗^	0.09	0.09
Sex^†^	0.06	−0.07	−0.06	−0.07
Relatives abroad^††^	0.12	0.12	−0.01	−0.01
Migrant relatives^††^	−0.05	−0.04	0.06	0.06
Frequent travels^††^	0.10	0.09	n/a	n/a
Genetic	−0.05	−0.05	−0.05	−0.05
Social constructivist	−0.01	−0.01	0.04	0.03
Multiculturalism	0.05	0.01	0.01	−0.01
National identity	−0.09	−0.09	−0.05	−0.05
Polyculturalism		0.18^∗∗^		0.09
*R*^2^	0.09	0.12	0.03	0.04
*F*	2.48^∗^	3.06^∗∗^	0.99	1.03
*df*	9, 233	10, 232	8, 242	9, 241
Δ*R*^2^		0.03		0.01
Δ*F*		7.65^∗∗^		2.00
*df*		1, 232		1, 241

*^∗^p < 0.05 and ^∗∗^p < 0.01. ^†^Male = 1 and Female = 2. ^††^No = 1 and Yes = 2.*

## General Discussion

The purpose of the two studies reported was to verify if polyculturalism would predict positive attitudes toward the continuing presence of former colonizers, and this relationship was studied in five cities in four Asian societies. The results in three of the five cities were consistent with the hypothesized relationship. That is, the participants from Hong Kong, Macau (both in Study 1) and Jakarta (Study 2) who endorsed polyculturalism were more likely to perceive the continuing presence of British, Portuguese, and Dutch businesses, media and arts, tourists, among others, respectively, as being good for their society. The relationship between polyculturalism and these positive attitudes were measured after controlling for a host of other variables that are typically associated with intergroup attitudes. These results extend the empirical evidence on the positive intergroup associations of polyculturalism by documenting its role in a specific form of intercultural contact that has not been previously studies.

That polyculturalism is positively associated with attitudes that relate to remnants of prior colonial relations is in a sense not surprising, as colonial societies can be viewed as examples of the mutual cultural connections in history. The colonial past may be viewed by some as a dark part of their history (cf., the violent oppression of Indonesians by the Dutch colonizers, Bijl, 2012), but for others the colonial past may be idealized (Musa, 2013; Trans, 2013; Lhatoo, 2015). There is generally a lot of ambivalence in people’s attitudes toward the colonial past, even among people from the country of colonizers (Cabecinhas and Feijó, 2010), and the results underscore how positive the intergroup consequences of the belief in polyculturalism can be. Our findings extend current evidence on the positive intergroup of polyculturalism to groups of people who may be linked to some fraught historical representations. Consistent with the theorizing on the epistemic function of lay theory of polyculturalism (Rosenthal and Levy, 2012), the belief about the mutual connection, and influence of cultures provides a positive lens with which the continuing presence of representatives of the former colonizer is perceived.

But the preceding discussion refers to the results from only three of the five cities; there were no significant associations between polyculturalism and the attitudes toward the continuing presence of former colonizers in Johor Bahru, Malaysia (Study 1) and Wonosobo, Indonesia (Study 2). We argued that the critical difference between the two groups of cities is the degree of intercultural contact within. Johor Bahru and Wonosobo were cities that did not experience high levels of intercultural contact, compared to Hong Kong, Macau, and Jakarta. We assumed that the level of intercultural contact is relevant in determining whether the lay theory of polyculturalism will be a meaningful belief system for the people in that society, that is, whether polyculturalism works as a lay theory. All lay theories effectively guide thoughts and actions only when the core ideas and beliefs are coherent knowledge representations and are accessible to the individuals as such – not merely as an isolated belief. The most potent lay theories are those for which the core ideas are regularly cognitively activated in the social environment (Hong et al., 2001). In such social environments, the core beliefs are chronically accessible and have the structure and function of a lay theory. In societies that do not experience much intercultural contact, like Johor Bahru and Wonosobo, the ideas that cultures are connected and mutually influencing each other may be meaningful in abstract sense, but may not function in a theory-like manner. People in those societies can have an understanding of how cultures may be dynamically connected, but that understanding may not be related to other social cognitive processes in a lay theory-like manner. Looking at the mean polyculturalism scores in the five cities, we see that the endorsement is relatively high in all cities; but the means may not reflect and endorsement of the lay theory as such, and instead might simply reflect endorsement of an isolated belief that does not relate to other intergroup processes.

In hindsight, one way of testing the differing functions of polyculturalism across the different societies would be to test whether the city moderated the relationship between polyculturalism and postcolonial attitudes in a hierarchical regression analysis. However, given that different that the measures in Study 1 involved different language versions of which there is no evidence of metric invariance, it was not prudent to do this moderation analysis in Study 1. But we attempted to test the moderation effect in Study 2 given that the scales in the two cities were in the same language. However, the interaction effect was not statistically significant (β = 0.122, *t* = 0.959, *p* = 0.338), most probably because the high multicollinearity with the interaction term (VIF = 9.432, tolerance = 0.11), which inflated the standard error term for the coefficient. Nevertheless, the results of the separate regression analyses provide evidence consistent with the notion that polyculturalism’s theory-like structure and function is best observed in societies with high levels of intercultural contact.

The preceding discussion underscores another contribution of the current study to the emerging research literature on polyculturalism. The empirical work on polyculturalism has so far focused on societies that experience substantial degrees of within-group and between-group intercultural contact as defined by Bochner (1982), where presumably there are many opportunities to engage the ideas of cultural connections and influence, and the core ideas that make up the lay theory of polyculturalism can take form. Our results suggest that this might be a necessary condition for observing the positive intergroup consequences of polyculturalism. If so, we should expect that the positive intergroup consequences associated with polyculturalism will not be observed in other societies with low levels of intercultural contact, and that means intergroup consequences that do not necessarily relate to colonial experiences. Future research should be designed to directly test this implication (see e.g., Tjipto and Bernardo, 2019).

Although the foregoing idea is consistent with theorizing on lay theories (Hong et al., 2001), it is also possible that high intercultural contact in and of itself is what makes people in that society more positively disposed to foreign groups in general, and representatives of the former colonizers in particular. This interpretation is consistent with the results of both Study 1 and 2 where the postcolonial attitudes in the high intercultural contact cities (Hong Kong, Macau, and Jakarta) seem to be numerically higher than those in the low intercultural contact cities (Johor and Wonosobo). Direct comparisons of means should be interpreted with caution because we cannot assume metric invariance across the different translations of the measures; but because the same measure was used in both Jakarta and Wonosobo, the results of Study 2 seem to make this alternative explanation more plausible. In our attempt to test the moderation effect in Study 2, the results indicated that when city and polyculturalism where in the regression model, polyculturalism still predicted postcolonial attitudes (β = 0.121, *t* = 2.792, *p* = 0.005), even as type of city (low or high intercultural contact) was stronger predictor (β = 0.246, *t* = 5.121, *p* < 0.001). The results do not complete rule out the alternative explanation, and future studies designed to directly measure and statistically control for individual and societal levels of intercultural experience are needed for this purpose. But there is some indication that polyculturalism contributes to postcolonial attitudes independently of the influence of intercultural contact. We note further, that level of intercultural contact may be confounded with other societal factors (e.g., urbanization, population density, societal level of educational attainment, and predominant political ideologies, etc.). So future research should be designed to control for these societal level differences, as well.

But one of the implications of the divergent findings related to level of intercultural contact is that the belief in polyculturalism is not necessarily enhanced in all postcolonial societies. It is possible to view a society’s colonial experience as an exemplification of the core ideas of polyculturalism; that is, the period in history where the foreign colonizers and the local population were directly in contact can be seen as an actual instance of the connection and mutual influence between cultures. As such, polyculturalism might be expected to be a well-formed belief system in postcolonial societies. The results of the two studies suggest that this is not the case. But the operative word in the previous statement is “history” as the participants in the study all did not have actual life experiences during the colonial period, and as such it seems that it is contemporary intercultural contact, and not historical intercultural contact that makes polyculturalism a salient lay belief in individuals.

Among the other limitations of our study, we acknowledge that the effect sizes of the significant effects found in the two studies were rather low, and this indicates that there are clearly other factors that shape attitudes toward the continuing presence of former colonizers. In the introduction we noted that people might differ in how the colonial experience is historically represented. It is worth noting, for example, that attitudes toward continuing colonial presence was positively associated with national identity in Macau participants, but not in any other sample; this suggests that for the Macau participants, the Portuguese presence in their society is associated with their national identity constructions. There are likely to be a host of other culture-specific factors that might predict attitudes toward the former colonizers in each postcolonial society. Even within one postcolonial society, individuals might vary significantly in how they construct this historical experience. In this regard, we note that the university students who participated in the study may not be very knowledgeable about the “colonizer” status of the countries referred to in the survey; they might have been thinking of these countries like any other foreign and that their presence in their society was just an expression of globalization. The results might be different if our participants were older adults in who lived during the colonial period in Hong Kong and Macau, or during the years after independence from the colonizers were established in Indonesia and Malaysia.

But the question of how representations of historical colonial experiences may shape notions of polyculturalism in postcolonial societies is an important one that cannot be addressed in the current study. To address this question, we need to inquire into individual’s own understanding of the historical postcolonial experience, whether this is viewed as positive, or negative. Interestingly, polyculturalism as a construct and the way it is operationalized in the scale is neutral with respect to the valence of the cultural connections and influence. Future theorizing about polyculturalism might consider looking at contrasting the positive and negative aspects of cultural connections.

## Conclusion

This study was conducted to explore whether belief in polyculturalism is associated with more positive attitudes toward the continuing presence of former colonizers in four postcolonial societies in Asia. Although there are limitations in the study, particularly with the small effect sizes and the use of only university student samples, the results extend the range of social psychological processes that seem to be positively associated with the lay theory of polyculturalism. Previous work has shown how polyculturalism predicts various forms of intergroup attitudes and processes in intercultural contexts; this study extends the evidence on the positive correlates of polyculturalism by showing positively associations with an external culture that is often characterized as having negative roles in one culture’s history. It also proposes and important caveat related to the positive outcomes associated with polyculturalism – that the lay theory seems to work only in societies where people have significant levels of intercultural contact. Finally, this study also offers a social psychological line of inquiry on postcolonial experiences, a topic that has not been extensively explored by psychologists but that is part of the everyday reality of people in most countries in Asia, Africa, and South America.

## Ethics Statement

The methods and materials used in this study were reviewed and approved by the University Research Ethics Committee, University of Macau; further review and approval of the procedures and materials was undertaken by some of the collaborating institutions. All participants gave their written informed consent before answering any part of the survey questionnaire, and consent forms kept separate from the survey questionnaires to ensure that no personal identifying information is included in the data for analysis.

## Author Contributions

AB conceptualized the research design, analyzed the data, led the writing of the manuscript, and agreed to be accountable for all aspects of the work in ensuring that questions related to the accuracy or integrity of any part of the work are appropriately investigated and resolved. MS contributed to data analysis and writing the manuscript. ST, BH, AK, and SY gathered the data and contributed to writing the manuscript.

## Conflict of Interest Statement

The authors declare that the research was conducted in the absence of any commercial or financial relationships that could be construed as a potential conflict of interest.
